# MRI Markers for Mild Cognitive Impairment: Comparisons between White Matter Integrity and Gray Matter Volume Measurements

**DOI:** 10.1371/journal.pone.0066367

**Published:** 2013-06-06

**Authors:** Yu Zhang, Norbert Schuff, Monica Camacho, Linda L. Chao, Thomas P. Fletcher, Kristine Yaffe, Susan C. Woolley, Catherine Madison, Howard J. Rosen, Bruce L. Miller, Michael W. Weiner

**Affiliations:** 1 Center for Imaging of Neurodegenerative Diseases, San Francisco VA Medical Center, San Francisco, California, United States of America; 2 Department of Radiology and Biomedical Imaging, University of California San Francisco, San Francisco, California, United States of America; 3 The Scientific Computing and Imaging Institute at the University of Utah, Salt Lake City, Utah, United States of America; 4 Department of Psychiatry, Neurology and Epidemiology, San Francisco VA Medical Center and University of California San Francisco, San Francisco, California, United States of America; 5 Department of Neurology, California Pacific Medical Center, San Francisco, California, United States of America; 6 Department of Neurology, University of California San Francisco, San Francisco, California, United States of America; Institution of Automation, CAS, China

## Abstract

The aim of the study was to evaluate the value of assessing white matter integrity using diffusion tensor imaging (DTI) for classification of mild cognitive impairment (MCI) and prediction of cognitive impairments in comparison to brain atrophy measurements using structural MRI. Fifty-one patients with MCI and 66 cognitive normal controls (CN) underwent DTI and T1-weighted structural MRI. DTI measures included fractional anisotropy (FA) and radial diffusivity (DR) from 20 predetermined regions-of-interest (ROIs) in the commissural, limbic and association tracts, which are thought to be involved in Alzheimer's disease; measures of regional gray matter (GM) volume included 21 ROIs in medial temporal lobe, parietal cortex, and subcortical regions. Significant group differences between MCI and CN were detected by each MRI modality: In particular, reduced FA was found in splenium, left isthmus cingulum and fornix; increased DR was found in splenium, left isthmus cingulum and bilateral uncinate fasciculi; reduced GM volume was found in bilateral hippocampi, left entorhinal cortex, right amygdala and bilateral thalamus; and thinner cortex was found in the left entorhinal cortex. Group classifications based on FA or DR was significant and better than classifications based on GM volume. Using either DR or FA together with GM volume improved classification accuracy. Furthermore, all three measures, FA, DR and GM volume were similarly accurate in predicting cognitive performance in MCI patients. Taken together, the results imply that DTI measures are as accurate as measures of GM volume in detecting brain alterations that are associated with cognitive impairment. Furthermore, a combination of DTI and structural MRI measurements improves classification accuracy.

## Introduction

Mild cognitive impairment (MCI) is a transitional stage between normal aging and early Alzheimer's disease (AD), though not all individuals with a diagnosis of MCI convert to AD [1]. Accurate identification of patients with MCI who are at increased risk of developing AD may facilitate timely initiation of treatments and possible prevention of dementia. While clinical assessments remain the standard diagnostic criteria for MCI, various imaging and biomarker measures have been proposed as complementary tests to improve diagnosis. Proposed measures include: MRI measured atrophy of the hippocampus and entorhinal cortex [2], 18F-fluorodeoxyglucose PET measured temporoparietal cortical hypometabolism, brain amyloid PET [3], and abnormal level of cerebrospinal fluid markers (amyloid-β42, total tau, and phosphorylated tau) [4], [5]. Among these proposed markers, MRI has attracted considerable interest, in part because of the low risk, non-invasive examinations and broad availability nowadays.

Aside from MRI measurements of brain atrophy [6]–[14], diffusion tensor imaging (DTI), a variant of MRI that is sensitive to microstructural tissue changes, has also shown promise in characterizing MCI [15]–[24]. DTI studies in MCI generally rely on diffusion summary measures, such as fractional anisotropy (FA), an index of the spatial directionality of tissue water diffusion and mean diffusivity (MD), a measure of the diffusion magnitude. These measures uniquely detect abnormalities in white matter (WM) regions. In particular, FA alterations in MCI – though often subtle [15], [17], [25]–[28] – have been found in limbic circuits (including posterior and parahippocampal tracts and fornix) [19], [29]–[32] and posterior callosal areas [33], [34]. To gain further insight into WM alterations in MCI, DTI studies have computed axial (DA) and radial (DR) diffusivity data in addition to FA. DR reflects the diffusion magnitude along the orthogonal directions of the diffusion tensor. In particular, an increase in DR has been associated with demyelination and axonal degeneration [35]. Some DTI studies using DR have demonstrated distinct zones of WM alterations in MCI where changes were barely seen with FA [22], [36]. However, aside from a previous work [19] in our laboratory, few studies [25], [37], [38] have compared the accuracy of DTI measurements with structural imaging measurements in characterizing brain alterations in MCI. It is not clear whether the various indices of DTI are superior to, or provide complementary information to structural MRI measures of brain atrophy for accurately classifying MCI and predicting cognitive deficits.

The aims of the study were: 1) to determine to what extent GM atrophy measured by structural MRI and WM alterations measured by DTI in prominent brain regions affected in AD are also associated with MCI; 2) to test whether WM alterations based on DTI improve the classification of MCI, in comparison to classifications based on GM volume; 3) to compare the accuracies of using GM volume and DTI measures for prediction of cognitive performance in MCI.

## Materials and Methods

### Subjects and Clinical Assessments

One hundred and seventeen subjects participated in the study. Subjects were recruited between May 2005 and October 2010 either by memory clinics in the San Francisco Bay Area, including the Memory Disorders Clinic at the San Francisco Veterans Affairs Medical Center, the Memory and Aging Center at the University of California, San Francisco, and the Memory Clinic at the California Pacific Medical Center or by posting flyers and advertisements in local newspapers. All subjects gave written informed consent, approved by the institutional review boards of the University of California San Francisco and the San Francisco VA Medical Center.

All subjects received the same research MRI examinations, medical and neurological examinations and neuropsychological testing. Domains assessed during baseline neuropsychological testing included: general cognitive ability (i.e., Mini-Mental State Examination, (MMSE) [39]), global cognitive functioning (i.e., Clinical Dementia Rating, (CDR) [40]), phonemic verbal fluency (D words) and semantic fluency (Animal Naming) [41] California Verbal Learning Test - Short Form (CVLT II) [42] including Immediate Recall (sum of trials 1–4 correct scores with total 36 items), 9-item Long Delay Free Recall and Long Delay Cued Recall. To facilitate predictions of accuracies, the 3 CVLT II recall scores (immediate recall, long free and cued recalls) were further composited as one CVLT score because they are correlated with each other.

None of the subject met the clinical diagnostic criteria for dementia. Fifty-four subjects were diagnosed with MCI by clinicians based on clinical criteria established by Peterson et al., or by the Alzheimer's Disease Cooperative Study (ADCS), or if the subject met the MCI core diagnostic features, which include: subjective memory complaints and memory difficulties that are qualified by an informant; memory impairment which exceeds normal aging, as indexed by low cognitive performance on one or more neuropsychological tests which assess learning or recall (for example, delayed recall, word list); a MMSE score greater than or equal to 24/30; a CDR score of 0.5 (but not demented); intact activities of daily living; and no dementia. All MCI subjects, with the exception of three whose diagnostic details were missing, were further divided into two groups: amnestic MCI (aMCI, if the MCI subjects had predominant memory impairments) and non-amnestic MCI (naMCI, if the MCI subjects had predominant non-memory impairments, such as executive, language, visual, behavioral, motor, or other deficits). Sixty-six cognitively normal subjects, whose neuropsychological scores were no worse than one standard deviation below the mean on standardized tests, but also not better than 2 standard deviations above the mean were included as controls. Exclusion criteria for all participants included any poorly controlled illness, use of medication or recreational drugs that could affect brain function, a history of brain trauma, brain surgery, ischemic events, or skull defects.

### MRI Acquisitions

All scans were preformed on a 4 Tesla (Bruker/Siemens) MRI system with a single housing birdcage transmit and 8-channel receiver head coil. T1-weighted images were obtained using a 3D volumetric magnetization prepared rapid gradient echo (MPRAGE) sequence with TR/TE/TI  = 2300/3/950 ms, 7-degree flip angle, 1.0×1.0×1.0 mm^3^ resolution, 157 continuous sagittal slices. In addition, FLAIR (fluid attenuated inversion recovery) images (TR/TE/TI  = 5000/355/1900 ms) and T2-weighted images (TR/TE = 4000/30 ms) were acquired for clinical reading. DTI was acquired based on a dual-refocused spin-echo EPI sequence supplemented with twofold parallel imaging acceleration (GRAPPA) [43] to reduce susceptibility distortions. Other DTI parameters were TR/TE  = 6000/77 ms, field of view 256×224 cm, 128×112 matrix size, yielding 2×2 mm^2^ in-plane resolution, and 40 contiguous slices each 3 mm thick. One reference image (*b* = 0) and six diffusion-weighted images (*b* = 800 s/mm^2^, along 6 noncollinear directions) were acquired.

### GM Volume Data Processing

Automated cortical volume measures, cortical parcellation, and subcortical segmentation were performed with FreeSurfer software package, version 4.5 (surfer.nmr.mgh. harvard.edu/fswiki).

For a full description of the FreeSurfer processing steps, see Fischl et al [44], [45]. In the first step, each T1-weighted volume was first corrected for head motion, then transformed into Talairach space using affine transformations (12 degrees of freedom), corrected for intensity non-uniformity, and separated from non-brain tissue. The remaining brain image volume was further normalized to match the intensity of a probabilistic atlas with 40 anatomical labeled regions of interest (ROI) per hemisphere before the atlas image was nonlinearly warped to the individual brain space to facilitate atlas-based tissue segmentation and anatomic labeling of subcortical structures, brain stem, cerebellum, and cerebral cortex. In the second step, FreeSurfer was used to generate topological cortical surface representations per hemisphere with theoretical surface bounded by the GM interface to white matter at the inner side and CSF at the outer side. Each hemisphere's cortical surface representation was mapped to the spherical coordinate system of the anatomical-labeled atlas, allowing an automated anatomical parcellation of cortex into gyral regions. The surface parcellation was then extended to GM volume, yielding parcellation of GM tissue sheet, volume and averaged thickness in each cortical and subcortical ROI. The anatomical accuracy of FreeSurfer processing was visually reviewed by trained readers with neuroanatomic knowledge but no corrections were performed by manual editing to avoid rater bias. Instead, data that did not pass the quality control of FreeSurfer processing were not included in the study.

**Figure 1:**
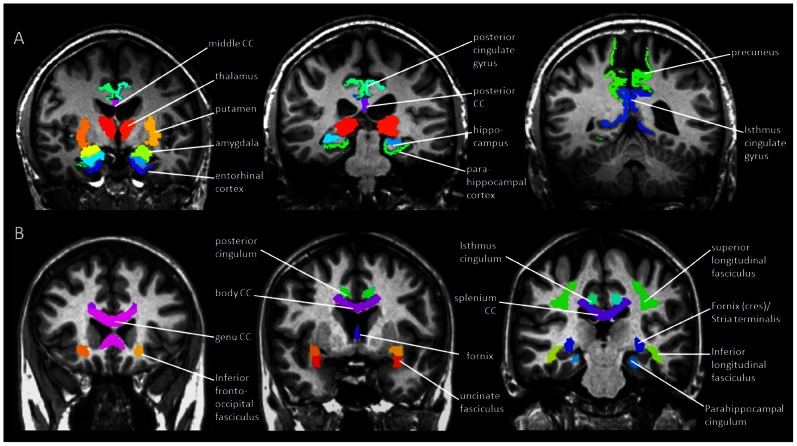
GM and WM parcellations. A. Automated parcellation of 21 cortical and subcortical ROIs for GM measurement, performed by Freesurfer software. B. Automated parcellation of 20 deep WM ROIs for DTI measurement, performed by SPM8.

Based on previous MRI reports of prominent brain alterations in AD and MCI [46]–[48], GM volumes were evaluated in 21 anatomical ROIs, including 3 callosal regions (anterior, middle and posterior corpus callosum), 8 medial temporal regions (bilateral hippocampus, entorhinal cortex, parahippocampal cortex and amygdala), 6 parietal regions (bilateral precuneus, posterior and isthmus cingulate gyrus) and 4 subcortical regions (bilateral thalamus and putamen) (Figure 1A). Although several other ROIs, such as inferior, middle and superior temporal cortex as well as inferior parietal cortex have been reported in the literature, we were unable to obtain reliable values from these regions across all subjects with the automated Freesurfer procedure due to limited image quality. Thus the study was limited in the above 21 ROIs in which FreeSurfer measurements were available for all participants. Regional values of the GM volume were normalized to total intracranial volume (ICV), to account for variations in head size.

### DTI Data Processing

Preprocessing of the DTI data included skull-striping, motion and eddy-current correction with FSL package (http://www.fmrib.ox.ac.uk/fsl/), and geometric distortion correction based on a variational matching algorithm [49]. After FA and DR images were created for each participant, these images underwent an automated region of interest (ROI) extraction procedure: 1) All FA images were resliced to the SPM8 white matter (WM) template using a rigid-body transformation (dimensions: 121×145×121 voxels, resolution: 1.5 mm^3^). 2) The ‘JHU ICBM-DTI-81’ atlas package (cmrm.med.jhmi.edu/), which includes a probabilistic FA atlas and anatomical labels of 44 deep WM ROIs from the DTI data of 81 subjects, was imported into SPM8, and also resliced to the SPM8 WM template using a rigid-body transformation. 3) The resliced FA images were warped nonlinearly to an intensity-averaged FA template generated from all subjects using a diffeomorphic registration algorithm (DARTEL). Consequently, all individual FA images and the ‘JHU ICBM-DTI-81’ FA and labeled atlas were spatially normalized onto a common space of the averaged FA template. 4) After that, a ‘reversed warping’ procedure was applied to assign the ‘JHU ICBM-DTI-81’ labeled atlas from the DARTEL common space to each individual FA space. 5) In subject's individual space, FA and DR were extracted from all labeled ROIs. To avoid inclusion of surrounding GM or CSF tissues, DTI values were obtained only in voxels with FA values greater than 0.20. Furthermore, data quality was reviewed and ROIs which contained visual mis-registration or WM hyperintensities were excluded. The decision to exclude ROIs with dominant WM hyperintensities was based on detection of a skewed FA distribution within the ROI toward very low FA values (<0.2) in combination with the visual inspection of the corresponding structural MRI for WM lesions.

Based on previous DTI reports [32], [50], FA and DR of 20 anatomical ROIs were measured, including 3 regions of the commissural tracts (genu, body and splenium of the corpus callosum), 9 regions of the limbic tracts (fornix and bilateral Fornix (cres) with Stria terminalis, posterior and isthmus cingulum, and hippocampal cingulum) and 8 regions of the association tracts (bilateral inferior and superior longitudinal fasciculi, inferior fronto-occipital fasciculi and bilateral uncinate fasciculi) (Figure 1B).

### Statistics

Differences between patients and controls were tested using a t-test for each ROI and MRI modality after effects of age and gender were linearly regressed out. For the rest of the analysis, FA, DR, and GM volume measures were centered and normalized to their respective standard deviations (i.e. transformed to z-scores) to eliminate scaling differences across the ROIs and MRI modalities. In addition, a non-parametric test using permutations of the diagnostic labels was applied to determine whether the heterogeneity of MCI has to be taken into account for findings of differences between uniform MCI and CN. The concept of false discovery rate (FDR) was used to account for the multiple comparisons problem [51]. An adjusted level of *p*<0.05 was selected as threshold of significance. The classification accuracy of FA, DR, and GM volume was tested using regularized logistic regressions with all respective ROIs included simultaneously as factors. An L2-norm regularization term (Ridge regression) was used to control for overfitting [52]. The classification accuracy of FA, DR, and GM were then compared pairwise by testing differences in the area under the curve of an operator characteristics analysis (ROC) based on bootstraping (using the roc.test function in the pROC package of R, URL: http://www.r-project.org/index.html). Similarly, to test the accuracy of FA, DR, and GM volume in predicting neurocognitive performance, regularized linear regressions were used with cognitive scores as outcomes and with all respective ROIs included simultaneously as factors. Accuracy was expressed as the root mean square error (RMSE) between predicted and clinical values, where a smaller RMSE indicating a more accurate prediction. Differences in RMSE distributions of FA, DR, and GM volume where compared using t-tests. Lastly, classifications and predictions were performed with 5-fold cross-validation and bootstrapping to estimate reliability in terms of 95% confidence intervals (CI).

## Results

### Demographic and Clinical Characteristics

The clinical and demographic characteristics of the subjects as well as global brain volumes are summarized by diagnostic group in Table 1. Differences in gender and years of education between MCI patients and CN subjects were not significant. However, MCI patients were older than CN subjects and performed worse on most neuropsychological tests as expected, with the exception of verbal fluency (D-words). The MCI patients had, on average, less total GM volume (normalized to ICV) than CN, while differences in total WM volume (also normalized to ICV) were not significant.

**Table 1:** Subjects and clinical characteristics.

	Control	MCI	MCI subtypes	
			naMCI	aMCI
Subject Number	66	51	17	31
Age (years)	67.2±10.0	72.8±8.7 †	71.9±10.9	73.1±7.5
Sex	**31 M ∶ 35 F**	**30 M ∶ 21 F**	**9 M ∶ 8 F**	**20 M ∶ 11 F**
Years of Education*	16.7±2.4	18.6±12.4	22.1±21.5	17.5±2.4
MMSE*	29.4±1.0	27.8±1.9 ‡	28.5±1.7	27.5±1.9
Immediate Recall (Trials 1–4)	28.2±4.1	19.1±6.8 ‡	21.6±6.0	17.1±6.8 †
Long Delay Free Recall	7.12±1.5	4.52±2.7 ‡	5.76±1.9	3.93±2.8 †
Long Delay Cued Recall	7.45±1.3	4.65±2.4 ‡	5.94±1.9	3.87±2.5 †
Verbal Fluency* (D-words)	14.6±5.6	13.4±6.0	14.3±5.9	12.9±6.1
Semantic Fluency* (Animal Naming)	21.6±6.1	15.7±6.2 ‡	18.9±7.0	13.9±5.3 †
ICV (cm^3^)	1076±125	1091±147	1046±111	1108±164
Total GM/ICV	0.40±0.04	0.37±0.04 †	0.38±0.03	0.36±0.04
Total WM/ICV	0.41±0.05	0.39±0.05	0.39±0.05	0.39±0.05

* 6 subjects' years of education was missing. 4 subjects' MMSE was missing. 28 subjects' verbal fluency and semantic fluency were missing. Note, smaller scores of neurocognitive measures indicate greater impairment.

Significance of group differences between paired groups (MCI vs. Control, aMCI vs. naMCI):

† 0.05<*p*≤0.001,

‡ *p*<0.001.

### Group differences based on GM volume and DTI measurements

**Figure 2:**
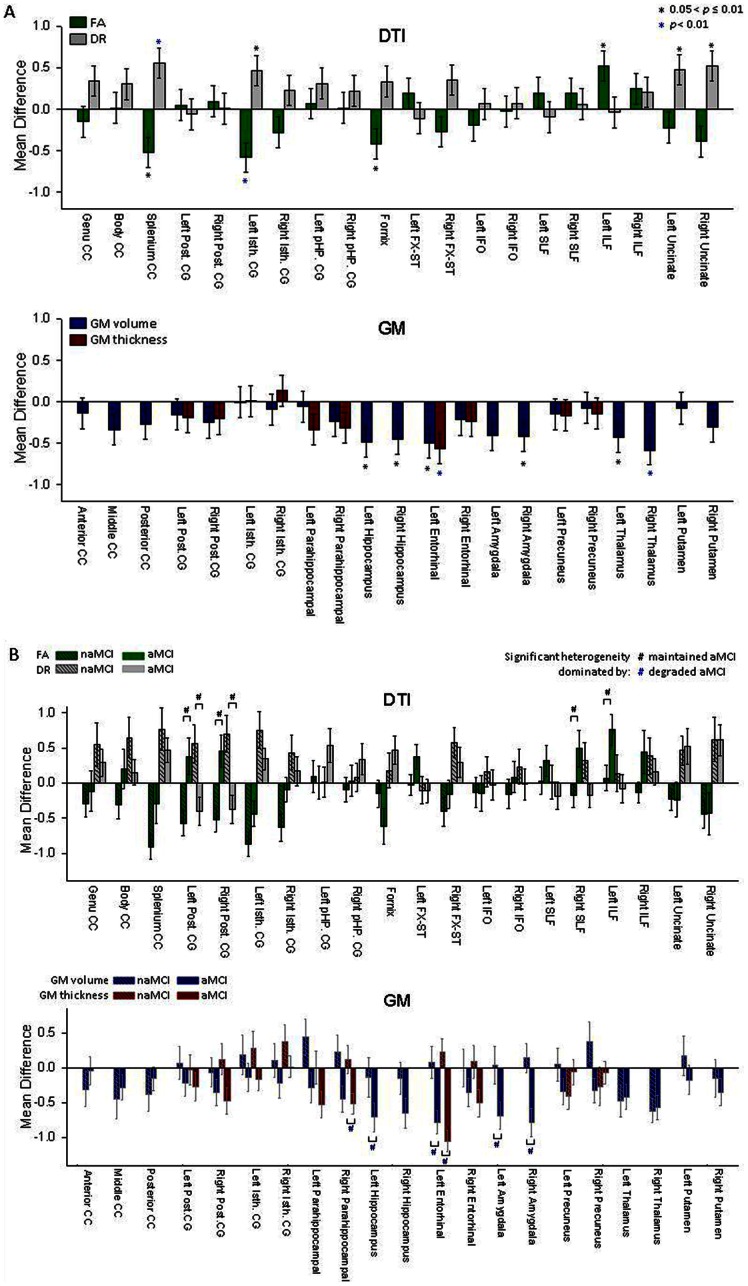
Mean differences of the DTI and GM measures between MCI and control. A. Mean differences and standard errors of regional DTI and GM measures, expressed as Z-scores, between MCI patients and controls for each ROI. Abbreviations:CC  =  corpus callosum; Post. CG  =  posterior cingulum; Isth. CG  =  isthmus cingulum; FX-ST  =  fornix (cres) and stria terminalis; IFO  =  inferior fronto-occipital fasciculus; SLF  =  superior longitudinal fasciculus; ILF  =  inferior longitudinal fasciculus. B. Mean differences and standard errors of regional DTI and MRI measures between aMCI, naMCI group and controls for each ROI. DTI and GM measures in ROIs with significant heterogeneities between aMCI and naMCI groups were labeled as “#”.

Mean and standard error of group differences in MRI measurements for all ROIs are depicted in Figure 2A, separately for diffusion and volume. As for DTI, mean FA was significantly reduced in MCI as compared to controls in the splenium of the corpus callosum, the left isthmus cingulum and the fornix. Interestingly, FA of the left inferior longitudinal fasciculus was increased in MCI. DR was increased in MCI as compared to controls in the splenium of the corpus callosum, the left isthmus cingulum and bilaterally in the uncinate fasciculi, consistent with reduced FA in these regions. As for GM volume, the most prominent volume losses in MCI patients compared to controls involved bilateral hippocampi, the left entorhinal cortex, the right amygdala, and the thalamus bilaterally. The most prominent regions of cortical thinning in MCI included the left entorhinal cortex. The effect sizes of significant group differences based on FA and DR were similar (effect size  = 0.42–0.61) to those based on GM volumes and thickness (effect size  = 0.42–0.61). Differences between the left and the right side of the brain were not significant (*p*>0.65).

Differences between MCI subgroups in MRI measurements for all ROIs are depicted in Figure 2B, separately for diffusion and volume. As for DTI, significant differences between the MCI subgroups were found in bilateral posterior cingulum, right superior longitudinal fasciculus and left inferior longitudinal fasciculus. In these regions, DTI (FA/DR) values of the naMCI group were more abnormal than those of the aMCI group, while the values of the aMCI group was on average not different from those of controls. The difference between the two MCI subgroups was dominated by better than normal values in the aMCI group. As for GM measures, significant differences between the MCI subgroups were found in the right hippocampus, left entorhinal cortex, bilateral amygdale, and the right parahippocampal cortex. In these regions, the difference between the two MCI subgroups was dominated by more GM loss and cortical thinning in the aMCI group. Hence, variations in the aMCI group dominated differences between MCI overall and controls.

### Relations between DTI and GM volume in MCI

**Figure 3:**
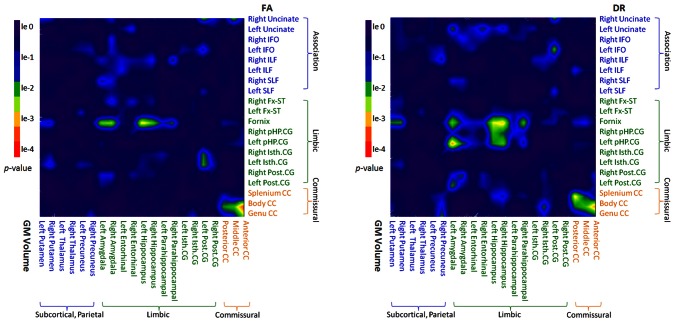
The correlations between DTI and GM volumes in MCI patients. The *p* value maps of the Pearson's correlation between DTI values and GM volumes. The green and warmer colors indicate significant correlations.

Relationships between regional DTI alterations in white matter and atrophy of GM in MCI were investigated using Pearson correlation coefficients. The significance of regional correlations are illustrate in Figure 3, separate for FA and DR measures in relation to GM volumes. Significant DTI-volume correlations were found between the limbic structures and the corpus callosum, whereas other regions showed no significant relationships.

### Group classifications

**Figure 4:**
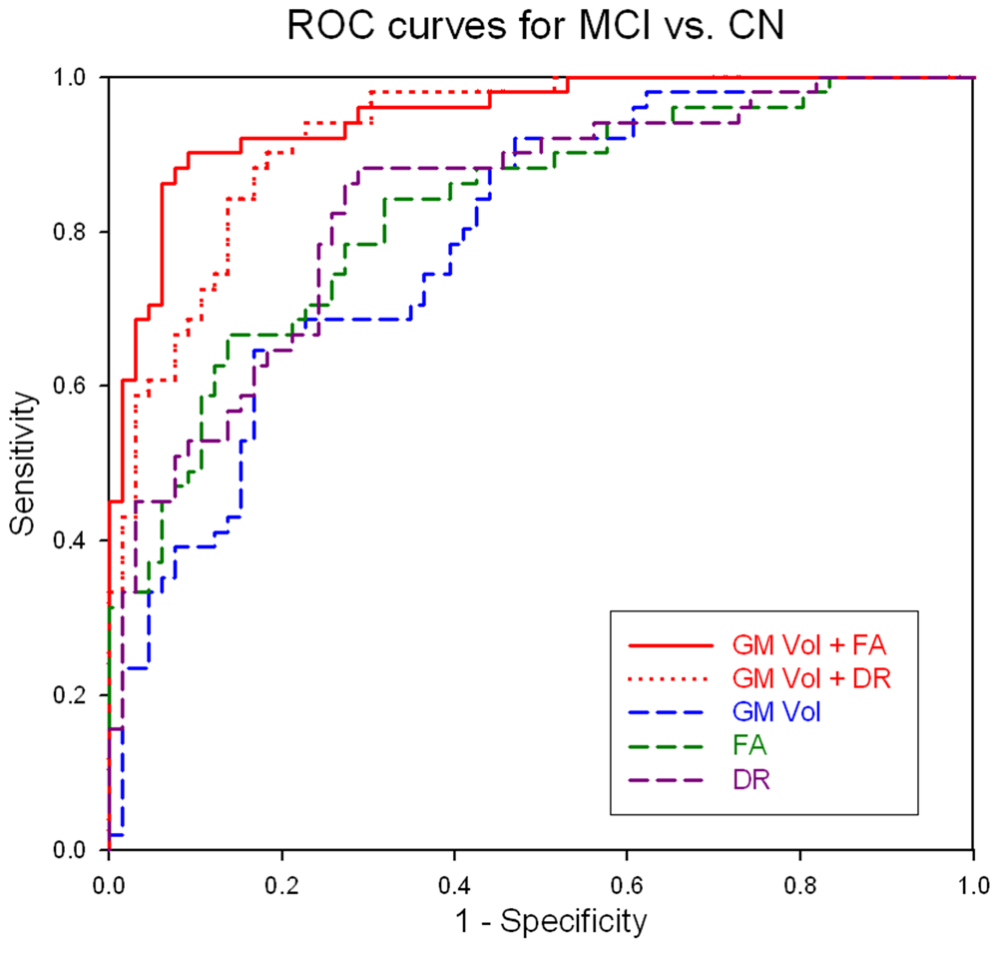
Classification accuracies based on DTI and GM volume measures. Receiver operator characteristic curves of classifications of MCI and control subjects based on DTI and GM volume measures used separately or together.

Classification accuracy of MCI and control subjects based on either FA, DR, GM volume alone or in combinations is summarized in Table 2. The corresponding receiver operating characteristic curves of each classification are illustrated in Figure 4. The table indicates that classifications based on FA or DR alone were significant (lower bound of the confidence interval >50%) in contrast to classifications based on GM volumes that were not better than chance. However, using either DR or FA together with GM volume further improved accuracy.

**Table 2:** Group classifications based on either DTI or GM volume measures separately or used together.

Measure	Sensitivity (%)	Specificity (%)	Accuracy (%)	Fitted AUC (%)	Cross-validated AUC (%)		
					(95% CI Lower)	median	(95% CI Upper)
FA	70.6	77.2	74.4	82.8	51.5	70.8	90.0
DR	66.7	78.8	73.5	83.5	58.0	78.5	95.0
GM volume	64.7	83.3	75.2	79.1	49.5	67.3	86.2
FA + GM volume	88.2	90.9	89.7	94.9	63.9	82.3^a^	96.2
DR + GM volume	82.3	86.4	84.6	92.6	61.9	81.2^b^	96.2

AUC – area under a receiver operating characteristic curve. AUCs were tested using bootstrap (2000 boots):

a Differences between modalities (i.e. FA + GM volume vs. GM volume) were significant at 95% confidence interval.

b Differences between modalities (i.e. DR + GM volume vs. GM volume) were significant at 90% confidence interval.

### Predictions of Cognitive Deficits

**Figure 5:**
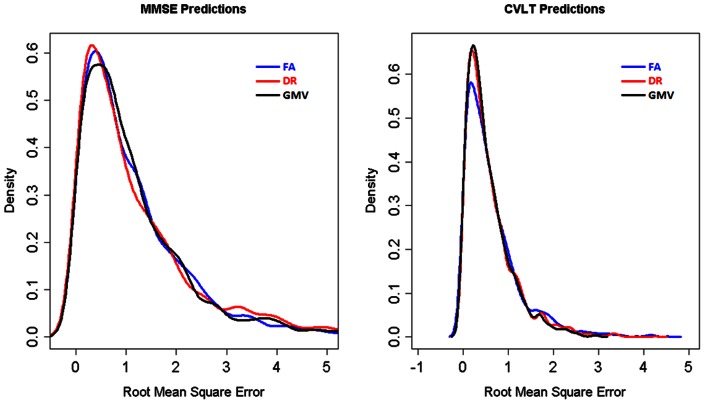
Accuracies of predicting cognitive scores based on DTI and GM volume measures. Distribution of root mean square errors in predicting MMSE and CVLT based on FA, DR or GM volume measures.

The accuracy of each FA, DR, or GM volume in predicting clinical scores of cognitive deficits is illustrated in Figure 5, showing the distribution of root mean square errors for each measure. The three imaging measures achieved similar levels of accuracy for predicting MMSE and CVLT.

## Discussion

The main findings of this study are: 1) MCI is associated with significant alterations of FA and DR as well as GM volume loss in specific regions, in agreement with previous findings. 2) FA and DR achieve better classification accuracy than GM volume alone, and using DTI and GM volume together further improves classification. 3) FA, DR and GM volume are similarly accurate in predicting cognitive performance. Taken together, the results imply that DTI of WM alterations is as accurate as structural MRI of GM volume in detecting brain alterations that associated with cognitive impairment. Furthermore, a combination of DTI and structural MRI measures improves accuracy.

DTI has been used in more than a hundred MRI reports of MCI and AD studies. However, the findings have been inconsistent [53], unlike structural MRI reports of medial temporal lobe atrophy in MCI and AD. Different methodologies in selecting regions of interest are a potential source of inconsistency in DTI findings. We used an automated region selection method to eliminate rater dependent bias and also evaluated all selected regions simultaneously for analysis to gain statistical power. Our findings of reduced FA and increased DR in the splenium, fornix isthmus cingulum, and uncinate fasciculi, are consistent with most other DTI studies with positive results [16], [17], [24], [28], [29], [31], [33], [54], [55]. However, our results conflict with some previous studies [15], [18], [25], [56]–[58] that found no significant DTI alterations in some of these regions. The findings also do not fully replicate our previous results [19] that showed significant FA reduction in the parahippocampal cingulum of MCI subjects. The inconsistent DTI findings might results partially from methodological differences between the studies. In particular, simply artifacts of microscopic heterogeneity, such as crossing fibers, may explain some of the inconsistencies. Moreover, heterogeneous pathological underpinnings of study populations may also result different DTI findings. For example, increasing evidences indicate that cognitively normal elderly individuals show pathological features of AD (i.e., neuritic plaques and neurofibrillary tangles). If DTI is sensitive to capture already relevant pathologies in cognitively normal group, the group differentiation with DTI might be diminished. Nonetheless, our findings of abnormal DTI in regions of the splenium, fornix, isthmus cingulum and uncinate fasciculus in MCI highlight a network of brain regions which are known to be vulnerable to AD pathology. The pattern of these regional DTI alterations suggests that regional degradation of WM matter integrity could be a sign of incipient AD pathology in MCI. An interesting finding is increased FA and decreased DR in the inferior longitudinal fasciculus in MCI. An increase in FA has been reported in other pathological conditions [59]. It has been proposed that reduced dendritic branching of the posterior parietal region may lead to an FA increase, though this hypothesis needs to be tested. However, increased FA could also be methodological artifacts induced by variations in the macroscopic architecture of the long association fibers [60]. The finding of increased FA of the inferior longitudinal fasciculus in MCI requires further investigations. In particular, DTI studies with more than six diffusion encoding directions need to be performed to achieve higher accuracy in measuring FA. In addition, the finding needs to be replicated in a separate population of MCI.

In contrast to DTI findings, our findings of GM atrophy in the medial temporal lobe and subcortical regions are generally in line with numerous other structural MRI studies of gray matter volume loss in MCI [6]–[12], [14]. As for the relationship between regional alterations in DTI and GM atrophy, significant correlations were limited to limbic structures and the corpus callosum. Although this suggests that microstructural degeneration and macroscopic tissue loss are correlated, the neural processes underlying the relationship between cortical atrophy and decreased white matter integrity remain unclear. Both retrograde and anterograde neuronal degeneration [61] could be responsible for the finding. Longitudinal studies are needed to investigate the causal relationship between white matter degradations and cortical atrophy in MCI and AD.

The finding that DTI outperformed structural MRI GM volume in the classification of MCI is surprising, because brain atrophy is generally considered to be strongly associated with cognitive impairments. Some MRI studies in MCI, using regional brain volumes reported between 73% to 91% classification accuracy [62]–[69]. Thus, the 79.1% fitted accuracy under the ROC curve for classifying MCI and healthy controls reported in our study still appears to be consistent with the results in literature. However, these previous studies employed mostly a single or a small number of ROIs, such as the hippocampus or the entorhinal cortex, achieving reasonably high sensitivity but lacking specificity potentially because atrophy of the hippocampus and/or entorhinal cortex are not features unique to MCI. By utilizing multiple brain regions simultaneously for classifications, we may have captured the heterogeneity of the MCI population better than evaluating each region separately. In contrast to studies that measured regional GM volumes, several DTI studies reported moderate classification accuracies (61–83%) of MCI [20], [37], [38], [70] based on a pre-selected single ROI. However, a complication in interpreting previous DTI results is that the pre-selected single ROI were not in consistent anatomical locations and the regional variations in DTI across different brain locations were not considered. In this study we attempted to take regional variations into account by using all selected ROIs simultaneously for classifications. Thus the results are less dependent on pre-selection of regions and potentially more powerful. Our classification results, when considering multiple regions, have shown similar cross-validated accuracies as that reported in previous DTI studies. The findings that DTI outperformed GM volumes over the cross-validated accuracies imply that DTI measurement is as accurate as the GM volume measurement in classifying MCI from healthy elderly controls. Furthermore, another potential strength of the DTI measurement is that DTI improves the diagnostic accuracies when using together with GM volume measures, this finding reconfirms our previous report and other reports in literature [19], [37], [38].

Our finding that DTI and GM volume measures were similarly accurate in predicting cognitive performance in MCI has not been previously reported. Generally, cognitive performance has been associated with gray matter atrophy and less with white matter alterations [71]–[74]. In particular, measuring hippocampal volume loss has often been used as predictor of memory deficits in MCI, consistent with well-known role of the hippocampus for memory consolidation [75]–[77]. Mean diffusivity of the parahippocampi [15] or the posterior cinguli [26] was reported predictive for memory performance in previous DTI studies with small samples. The abnormalities reported by DTI may reflect WM insults such as myelin/axonal degeneration, oligodendrocytes death, reactive gliosis, and small infarctions that may occur secondarily to volume loss, which is known to be associated with memory deficits. Consequently, it is reasonable to consider DTI as a possible predictor to represent simultaneous though not identical neuropathological processes in addition to using GM volume. Although we cannot determine in this cross-sectional study whether the predictions based on DTI reflect primary and secondary effects, our results imply that alterations in WM are similarly important for predictions of cognitive performance compared to GM volume loss. Moreover, by taking regional variations into account, we reduced bias toward localized alterations that may not be characteristic for MCI. We believe that our approach is more reliable and also provides more interpretable results than testing a few pre-determined regions separately. Our finding that DTI achieved similar power as GM volumes in predicting cognitive deficits highlights the potential value of DTI.

Our study has several limitations. It is possible that vascular factors contributed to FA, DR, and GM volume differences between MCI and controls [34], [78], although we avoided brain areas with visual WM hyperintensities when selecting ROIs. On the other hand, some particular ROIs may include surrounding voxels that contain GM, CSF and crossing fibers that may have exaggerated group differences of DTI measures. Although in our study DTI measures were limited in ROIs that contain voxels of FA >0.2, there is no definition of a fiber boundary that enables fully exclusion of partial volume. The potential influence of vascular factors and partial volume effects on our results requires further investigation but is beyond the scope of this study. Second, the MCI group included not only subjects with memory complaints (amnestic MCI), but also those with executive, language or other deficits (non-amnestic MCI) as the dominant clinical symptoms. Correspondingly, the group may also be heterogeneous with respect to the pathological underpinnings of the clinical features. Furthermore, the number of subjects in this cohort, who converted to AD after 2-years clinical follow-up was too small to determine whether the DTI and GM volume alterations were linked to a conversion to AD with a reasonable level of confidence. Studies with longer follow-ups are needed to compare the predictive value of structural MRI and DTI for the development of AD. A methodological limitation was that we did not capture complete distributions of DTI and GM volume variations across the whole brain. This may have biased our results as well as reduced sensitivity as potential variations outside the regions of interest were ignored. Although recently advances have been made in processing all imaging voxels simultaneously, using machine learning algorithms [79], the methodological complexity of machine learning for MRI is not yet fully understood, especially when multiple imaging variables are used together. As machine learning methodologies in MRI become more robust, the comparisons between DTI and structural MRI should be repeated. Another methodological limitation is that DTI was performed with only 6 diffusion gradient directions, while more directions are known to stabilize tensor estimations. However, at the time this study was started, a DTI protocol with more diffusion directions was not available. It is therefore possible that our DTI results are confounded by larger error margins than recent DTI studies using more directions.

In conclusion, this study shows that DTI measures are as accurate as measures of GM volumes in characterizing brain alterations associated with cognitive impairment, and a combination of DTI and structural MRI measurements improves classification accuracy.
